# Predictive Model and Software for Inbreeding-Purging Analysis of Pedigreed Populations

**DOI:** 10.1534/g3.116.032425

**Published:** 2016-09-06

**Authors:** Aurora García-Dorado, Jinliang Wang, Eugenio López-Cortegano

**Affiliations:** * Departamento de Genética, Universidad Complutense, Madrid 28040, Spain; † Institute of Zoology, Zoological Society of London, London NW1 4RY, United Kingdom

**Keywords:** inbreeding depression, purging coefficient, rate of inbreeding depression, inbreeding load, logarithmic fitness

## Abstract

The inbreeding depression of fitness traits can be a major threat to the survival of populations experiencing inbreeding. However, its accurate prediction requires taking into account the genetic purging induced by inbreeding, which can be achieved using a “purged inbreeding coefficient”. We have developed a method to compute purged inbreeding at the individual level in pedigreed populations with overlapping generations. Furthermore, we derive the inbreeding depression slope for individual logarithmic fitness, which is larger than that for the logarithm of the population fitness average. In addition, we provide a new software, PURGd, based on these theoretical results that allows analyzing pedigree data to detect purging, and to estimate the purging coefficient, which is the parameter necessary to predict the joint consequences of inbreeding and purging. The software also calculates the purged inbreeding coefficient for each individual, as well as standard and ancestral inbreeding. Analysis of simulation data show that this software produces reasonably accurate estimates for the inbreeding depression rate and for the purging coefficient that are useful for predictive purposes.

Due to the increase in the frequency of homozygous genotypes for (partially) recessive deleterious alleles under inbreeding, inbreeding depression for fitness is a major threat to the survival of small populations (Falconer and Mackay 1996; Saccheri *et al.* 1998; Hedrick and Kalinowski 2000; Frankham 2005). However, as these alleles become more exposed under inbreeding, an increase in the efficiency of natural selection against them is also expected, which is known as genetic purging and tends to reduce the frequency of deleterious alleles, and, consequently, the fitness decline induced by inbreeding (Templeton and Read 1984; Hedrick 1994; Ballou 1997; García-Dorado 2012, 2015).

The first models developed to detect the consequences of purging on inbreeding depression from pedigree data accounted for purging by using an ancestral purging coefficient, *F_a_*, that represents the proportion of an individual’s genome that is expected to have been exposed to homozygosis by descent in at least one ancestor (Ballou 1997; Boakes and Wang 2005). The rationale is that, due to genetic purging, inbred individuals with inbred ancestors would have fewer deleterious alleles than individuals with the same inbreeding but noninbred ancestors.

More recently, a theoretical Inbreeding-Purging (IP) approach has been developed that predicts the evolution of fitness under inbreeding by taking purging into account by means of a purged inbreeding coefficient *g*. This IP model considers that purging acts against a purging coefficient (*d*) that quantifies the component of the deleterious effects that are expressed only under inbreeding (García-Dorado 2012). For a single locus model, *d* represents the per copy excess of the deleterious effect in the homozygous over that expected on an additive hypothesis, and its value ranges from *d* = 0 (no purging) to *d* = 0.5 (purging against recessive lethal alleles). In practice, as *d* varies across loci, a single value, known as the effective purging coefficient (denoted by *d_e_* in García-Dorado 2012; here denoted by *d* for simplicity), can be used to compute approximate predictions for the overall consequences of purging over the whole genome. Estimating this effective *d* value is of main interest as it will provide a measure of the purging occurred, and will allow us to use the model to predict the expected evolution of fitness.

Until now, the only empirical estimates of the purging coefficient *d* have been obtained from the evolution of fitness average in *Drosophila* bottlenecked populations (Bersabé and García-Dorado 2013; López-Cortegano *et al.* 2016). However, in conservation practice, fitness data are often available for pedigreed populations. Two versions of the IP model were originally proposed, one aimed to predict mean fitness as a function of the number of generations under a reduced effective population size *N_e_*, the other one aimed to predict individual fitness from pedigree information. Nonetheless, the latter version was developed only for data with nonoverlapping generations, which imposes serious limitations to its use in experimental and conservation practice.

Here we extend the IP model to compute the purged inbreeding coefficient *g* for individuals in pedigrees with overlapping generations. Furthermore, we derive a new expression that gives the expected individual log-fitness as a function of *g*, and of the initial inbreeding load *δ*, deriving the slope of inbreeding depression for individual logarithmic fitness, which is larger than that for the logarithm of average population fitness. In addition, we present the new free software PURGd, based on this IP approach, that is able to use data for fitness traits in pedigreed samples to test for purging, and to estimate the corresponding effective purging coefficient *d*. This software also estimates the inbreeding depression rate for individual fitness, and computes the standard (*F*), ancestral (*F_a_*), and purged (*g*) inbreeding coefficients for the pedigreed individuals.

## Methods

### The Model

#### The rate of inbreeding depression estimated from individual fitness:

In order to analyze and interpret the consequences of inbreeding and purging at an individual level, we must first consider the relationship between individual fitness and inbreeding in a neutral model with no natural selection.

Assume a population where a number of deleterious alleles segregate at a low frequency, *q*, at different loci acting multiplicatively on fitness. From here onwards we will concentrate just on (partially) recessive deleterious alleles, which are assumed to be responsible for inbreeding depression. Each locus has two alternative alleles, the wild one and the mutant deleterious allele. It has three genotypes, with average fitness 1, 1-*hs*, and 1-*s* for the wild homozygous genotype, the heterozygous genotype, and the deleterious homozygous genotype, respectively. Therefore, the population inbreeding load, which can be measured by the number of lethal equivalents (Morton *et al.* 1956), is

δ=∑2dq(1−q), (1)

where *d* = *s*(1/2−*h*), and the sum is over all the relevant loci.

For simplicity, we will assume that the initial frequency of each deleterious allele is small enough that homozygous genotypes are produced only due to inbreeding. Furthermore, in this section, we will also assume completely recessive gene action (*h* = 0; *s* = *2d*). This assumption smooths the explanation below, but is not necessary for the validity of the conclusions.

After some inbreeding, the fitness of an individual that is homozygous by descent for deleterious alleles at *n* loci is

$$W=W_{\max}\left(1-\varepsilon \right){\left(1-2d\right)}^{n},$$ (2)

where *W*_max_ is the maximum possible fitness value, and *ε* is the proportional reduction of the fitness of that individual due to all kinds of environmental and genetic factors, excluding inbreeding depression.

If the inbreeding load is due to many loosely linked deleterious loci, and deleterious alleles segregate at low frequency, the number, *n_i_*, of deleterious alleles in homozygosis for an individual *i* with standard Wright’s inbreeding coefficient *F_i_* should be Poisson distributed. Since the probability of being homozygous for a deleterious allele in noninbred individuals is assumed to be negligible, the expected value of this number should be *E*(*n_i_*) = ∑ *F_i_ q*(1−*q*) (Falconer and Mackay 1996). Thus, substituting ∑*q*(1−*q*) from Equation (1), we obtain that the mean of this Poisson distribution is

$$\lambda =E\left(n_{i}\right)=F_{i}\delta /2d.$$ (3)

Therefore, from Equation 2, and assuming that *ε* and *F* are independent, the expected fitness of an individual *i* that has genealogical inbreeding *F_i_* is

$$E\left(W_{i}\right)=E\left(W_{0}\right)\sum_{n=0}^{\infty }\frac{e^{-\lambda }\lambda^{n}}{n!}{\left(1-2d\right)}^{n}$$

where *E*(*W*_0_) = *E*[*W*_max_(1−*ε*)] is the expected fitness of a noninbred individual. The equation above can be rewritten as

$$E\left(W_{i}\right)=E\left(W_{0}\right)e^{-\lambda 2d}\sum_{n=0}^{\infty }\frac{e^{-\lambda }\lambda^{n}\;e^{\lambda 2d}}{n!}{\left(1-2d\right)}^{n},$$

and can be rearranged as

$$E\left(W_{i}\right)=E\left(W_{0}\right)e^{-\lambda 2d}\sum_{n=0}^{\infty }\frac{\;e^{-\lambda \left(1-2d\right)}{\left[\lambda \left(1-2d\right)\right]}^{n}}{n!}.$$

Noting that $\overset{\infty }{\underset{n=0}{\sum }}e^{-\lambda \left(1-2d\right)}{\left[\lambda \left(1-2d\right)\right]}^{n}/n!$ adds up all the probabilities for a Poisson distribution with mean *λ*(1−2*d*) (*i.e.*, it equals 1), and, since *λ* = *F_i_* *δ*/2*d* (Equation 3), we obtain the exponential expression

$$E\left(W_{i}\right)=E\left(W_{0}\right)\;e^{-\delta F_{i}},$$ (4)

and, similarly, the average fitness of a population with average inbreeding *F_t_* in generation *t*, as far as the number of loci homozygous for a deleterious allele per individual can be assumed to be Poisson distributed with mean *λ* = *F_t_* *δ*/2*d*, is

$$E\left(W_{t}\right)=E\left(W_{0}\right)\;e^{-\delta F_{t}},$$ (5)

In order to estimate *δ* from observed inbreeding depression, logarithms are usually taken in Equation 4 or Equation 5 to obtain a linear model of the kind ln(*W*) = ln(*W*_0_)−*δ* *F*. However, since the average of the logarithms of a variable is smaller than the logarithm of the average (see Jensen’s inequality), applying this procedure to individual fitness values can produce large upwards bias in the estimate of *δ*. Thus, from Equation 2, the logarithm of fitness (log-fitness hereafter) for an individual that is homozygous by descent for *n* deleterious alleles is

$$\ln\left(W\right)=\ln\left[W_{\max}\left(1-\varepsilon \right)\right]+\ln\left[{\left(1-2d\right)}^{n}\right],$$

so that, using the Poisson distribution of *n_i_*, the expected value for log-fitness for an individual *i* that has genealogical inbreeding *F_i_* is

$$E\left[\ln\left(W_{i}\right)\right]=E\left[\ln\left(W_{0}\right)\right]+\sum_{n=0}^{\infty }\ln\left[{\left(1-2d\right)}^{n}\right]\;\frac{e^{-\lambda }\lambda^{n}}{n!},$$ (6)

where the intercept *E*[ln(*W*_0_)] = *E*{ln[*W*_max_ (1−*ε*)} represents the average of individual log-fitness at the noninbred population. Since the second term equals ln(1−2*d*)*E*(*n_i_*), using Equation 3, Equation 6 gives

$$E\left[\ln\left(W_{i}\right)\right]=E\left[\ln\left(W_{0}\right)\right]+\frac{\ln\left(1-2d\right)}{2d}\delta F_{i}.$$ (7)

On the other hand, in agreement with classical theory (Morton *et al.* 1956), Equation 4 and Equation 5 imply

$$\ln\left[E\left(W_{i}\right)\right]=\ln\left[E\left(W_{0}\right)\right]-\delta F_{i}$$ (8)

and

$$\ln\left[E\left(W_{t}\right)\right]=\ln\left[E\left(W_{0}\right)\right]-\delta F_{t}$$ (9)

It is interesting to note that, as indicated by Morton *et al.* (1956), the two equations above produce good approximation in so far as each individual locus makes a small contribution to the overall expected inbreeding load.

Equation 8 allows *δ* to be estimated from the decline in average fitness for a given inbreeding level, as in designs where fitness is measured in a sample of outbred and a sample of inbred individuals (for example, full sib offspring). Equation 9 allows *δ* to be estimated, generally using linear regression, from the decline in average fitness through generations of inbreeding, as in a population that has experienced a reduction in size. Both approaches induce no bias in the estimate of *δ*, in so far as natural selection can be ignored and sample sizes are sufficiently large that the expected value of the logarithm of the sample’s average is close to the logarithm of the expected average {*i.e.*, to ln[*E*(*W_t_*)] or ln[*E*(*W_i_*)]}.

However, Equation 5 shows that the slope of linear regression for the logarithm of individual fitness on individual inbreeding is

$$b=\;\frac{\ln\left(1-2d\right)}{2d}\delta ,$$ (10)

where the limit of [ln(1−2d)]/2d as *d* approaches 0 is −1. Therefore, unless *d* is very small, −*b* provides an upwardly biased estimate for the inbreeding load *δ*.

Here, we present a software package (PURGd) that estimates the purging coefficient and the inbreeding load from the relationship between individual fitness and individual inbreeding using two alternative approaches. The first approach estimates *b* from the linear regression of log individual fitness on individual genealogical inbreeding. The second approach estimates *δ* by numerical least squares (LS) from untransformed fitness, directly using Equation 4. In addition to allowing the use of individual fitness data including 0 values (as in the case of a dichotomous 0–1 variable for dead-alive records), this procedure allows direct estimation of *δ*, instead of *b*.

### The inbreeding-purging (IP) model: computing purged inbreeding and purged coancestry from pedigrees

According to the IP approach, in order to incorporate the consequences of purging, the evolution of fitness under inbreeding should be predicted by replacing the standard inbreeding coefficient, *F*, with a purged inbreeding coefficient, *g*, where *F* is weighted by the reduction in frequency of deleterious alleles induced by purging. Thus, Equation 4 and Equation 5 become:

$$E\left(W_{i}\right)=E\left(W_{0}\right)\;e^{-\delta g_{i}}.$$ (11)

$$E\left(W_{t}\right)=E\left(W_{0}\right)\;e^{-\delta g_{t}}.$$ (12)

García-Dorado (2012) derived equations allowing to compute *g_i_* for individuals in pedigrees with nonoverlapping generations. These *g_i_* values depend on the pedigree and on the *d* value defined above as *d* = *s*(1/2−*h*), which here represent the purging coefficient. For multilocus models where *d* varies across loci, it has been shown empirically using extensive simulations that *d* can be replaced with an effective purging coefficient that accounts for purging across the whole genome to a good approximation. This effective purging coefficient was denoted *d_e_* in García-Dorado (2012) but here, for simplicity, it will be denoted *d* and referred to just as purging coefficient.

In what follows, we derive more general expressions to compute approximate *g_i_* values for individuals in arbitrary pedigrees that can include overlapping generations.

**Figure 1:**
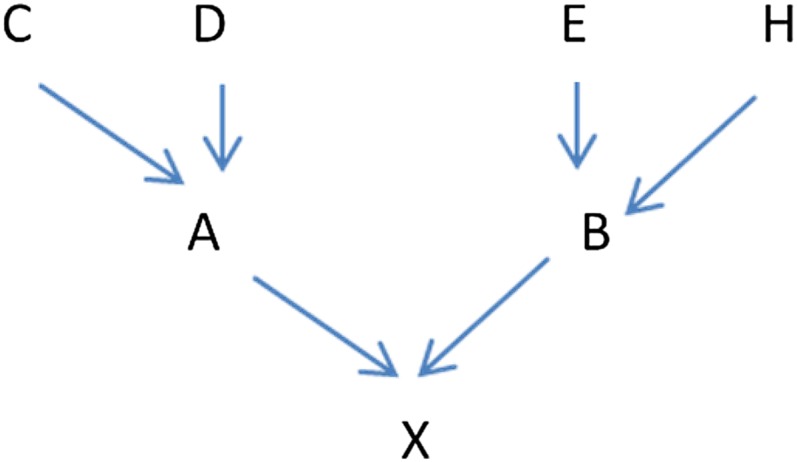
General pedigree notation.

The purged inbreeding coefficient *g_i_* is defined as *g_i_* = *E*(*F_i_* *q_i_*)/*q*_0_ , where *E* stands for “expected value” and *q*_0_ (*q_i_*) is the frequency of the deleterious alleles in the base population (expected in individual *i*). In other words, (*q*_0_ *g_i_*) is the probability that individual *i* is homozygous by descent for the deleterious allele. In order to settle notation, we will use A and B to denote individual X’s parents, C and D to denote individual A’s parents, and E and H to denote individual B’s parents, as shown in Figure 1.

Let *f*(A,B) be Malécot’s coancestry between individuals A and B; *i.e.*, the probability that a random allele from a neutral locus in A, and, independently, a random allele from the same locus in B, are identical by descent (IBD) (Malécot 1948). By analogy to García-Dorado (2012), we will assume that the probability that two copies sampled from different individuals are IBD is unaffected by the fitness values of the copies.

As in García-Dorado (2012), let *γ*(A,B) be the purged coancestry between A and B, which are assumed to have survived purging selection. In other words, [*q*_0_ *γ*(A,B)] is the probability that two alleles, one randomly sampled from A, and the other independently sampled from B, at the same locus, are IBD for the deleterious allele. Therefore, the purged inbreeding coefficient for an individual X that has still not undergone purging, can be computed as the purged coancestry between their parents; *i.e.*, *g_x_* = *γ*(A,B).

Note that *q*_0_ · *γ*(A,B) could be defined as the probability that an allele sampled randomly from A is deleterious and IBD to another allele sampled randomly from B, *i.e.*, *q*′_A_ *f*(A,B), where *q*′_A_ denotes the frequency of the deleterious allele in individual A conditional to it having survived purging selection. Alternatively, [*q*_0_ · *γ*(A,B)] could also be defined as the probability that an allele sampled randomly from B is deleterious and IBD to an allele sampled randomly from A, *i.e.*, *q*′_B_ *f*(A,B). Therefore, by averaging both alternatives we obtain

$$q_{0}\cdot \gamma \left(A,B\right)=1/2\left(q_{A}^{\prime }+q_{B}^{\prime }\right)f\left(A,B\right)$$ (13)

Finally, let *γ*(A,B|E) be the purged coancestry between A and B conditional to sampling from B the copy inherited from E. In other words, [*q*_0_ *γ*(A,B|E)] is the probability that one allele sampled randomly from A is deleterious and IBD to the copy that B inherited from E.

Therefore, [*q*_0_ · *γ*(A,B)] is the probability that the copy sampled from B was inherited from E (*i.e.*, 1/2), and then the two copies (one sampled from A and the other one from B) are IBD for a deleterious allele, plus the analogous probability corresponding to sampling from B the copy inherited from H, *i.e.*:

$$q_{0}\;\gamma \left(A,B\right)=1/2\;q_{0}\;\gamma \left(\mathrm{A,B}|E\right)+1/2\;q_{0}\;\gamma \left(\mathrm{A,B}|H\right)$$ (14)

Thus, we need a systematic procedure to compute *γ*(A,B|E) that can be used recurrently to obtain *γ*(A,B) and *g_x_*. To achieve this, we note that the probability that one allele randomly sampled from A and the copy that B inherited from E are IBD for the deleterious allele can be computed in two ways:

1. After B survives purging, the copy in B inherited from E is the deleterious allele. Since purging is expected to reduce deleterious frequency in B by a factor (1−2*d* *F*_B_) (García-Dorado 2012), this occurs with probability *q*′_E_ · [1−2·*d*·*F*_B_]. Furthermore, this copy is IBD to that sampled from A. Taking into account that *f*(A,E) is assumed to be independent on the allelic state (*i.e.*, is the same for deleterious as for wild alleles), this occurs with probability *q*′_E_ · [1−2·*d*·*F*_B_] · *f*(A,E).
2. The copy sampled from A is deleterious and is IBD to the copy that B has inherited from E. This occurs with probability *q*′_A_ · *f*(A,E)

Thus, we compute [*q*_0_ · *γ*(A,B|E)] by averaging these two probabilities above, which gives

$$q_{0}\cdot \gamma \left(A,B|E\right)=1/2\left(q_{A}^{\prime }+q_{E}^{\prime }\right)f\left(\mathrm{A,E,}\right)-q_{E}^{\prime }\cdot f\left(\mathrm{A,E,}\right)d\cdot F_{B}.$$ (15)

Now, if inbreeding progresses slowly, the last *q*′_E_ in the above expression can be replaced with 1/2 (*q*′_A_ + *q*′_E_) to a good approximation, and Equation 15 approaches

$$q_{0}\cdot \gamma \left(\mathrm{A,B}|E\right)\approx \left[1/2\left(q_{A}^{\prime }+q_{E}^{\prime }\right)f\left(A,E\right)\right]\left(1-d\cdot F_{B}\right),$$ (16)

which, applying Equation 13 to A and E, gives the approximate expression

$$\gamma \left(A,B|E\right)=\gamma \left(A,E\right)\left(1-d\cdot F_{B}\right)$$ (17)

Therefore, substituting the conditional purged coancestry given by Equation 17 into Equation 14, we obtain

$$\gamma \left(A,B\right)=1/2\left[\gamma \left(A,E\right)+\gamma \left(A,H\right)\right]\left(1-d\cdot F_{B}\right).$$ (18)

As in the case of classical Malécot’s coancestry (*f*), purged inbreeding arises from the pedigree knots where *γ*(A,B) happens to represent a self-coancestry (A and B are the same individual). In those cases, as previously shown (García-Dorado 2012),

$$\gamma \left(\mathrm{A,A}\right)=1/2\left[\text{1+}g_{A}\right]\left[1-2d\;F_{A}\right].$$ (19)

Equation 18 is analogous to the classical recurrent expression that gives the coancestry between A and B as the average coancestry between A (which should not be younger than B) and both parents of B {*f*(A,B) = [*f*(A,E) + *f*(A,H)]}, except that Equation 18 accounts for the purging occurred in B. Thus, Equation 18 can be used recurrently together with Equation 19 to compute purged coancestry between pairs of individuals that have survived purging, which equates the purged inbreeding expected for their offspring [*g_x_* = *γ*(A,B)].

To compare this approach with that previously derived for nonoverlapping generations, we note that, analogously to Equation 18, we can write

$$\gamma \left(\mathrm{A,E}\right)=1/2\left[\gamma \left(C\text{,}E\right)+\gamma \left(\mathrm{D,E}\right)\right]\left(1-d\;F_{A}\right),$$ (20)

and

$$\gamma \left(\mathrm{A,H}\right)=1/2\left[\gamma \left(C\text{,}H\right)+\gamma \left(\mathrm{D,H}\right)\right]\left(1-d\;F_{A}\right).$$ (21)

And, substituting Equation 20 and Equation 21 into Equation 18, we obtain

$$\gamma \left(A,B\right)=1/4\left[\gamma \left(C\text{,}E\right)+\gamma \left(D,E\right)+\gamma \left(C,H\right)+\gamma \left(D,H\right)\right]\left(1-d\;F_{A}\right)\left(1-d\;F_{B}\right).$$

This expression slightly overrates the purged coancestries (and, therefore, the purged inbreeding coefficients) derived by García-Dorado (2012) for nonoverlapping generations, which gave

$$\gamma \left(A,B\right)=1/4\left[\gamma \left(C\text{,}E\right)+\gamma \left(D,E\right)+\gamma \left(C,H\right)+\gamma \left(D,H\right)\right]\left[1-d\left(F_{A}+F_{B}\right)\right]$$ (22)

The overrate is due to the use of the approximation *q*′_E_ ≈ 1/2 (*q*′_A_ + *q*′_E_) to derive Equation 16, which, on average, underrates the deleterious frequency against which purging is operating. The bias should, however, be small, since the squared term ($d^{2}$ *F*_A_ *F*_B_) can be important only where *d* and *F* values are large, which implies small *γ* and *g* values. Using simulated pedigrees in bottlenecked populations with nonoverlapping generations, we found that the correlation between *γ*(A,B) computed from Equation 18 and from García-Dorado (2012) was always larger than 0.999 for a wide range of different purging coefficients from *d* = 0 to *d* = 0.5 (results not shown).

Finally, it must be noted that, for IP predictions to be reliable, drift should be relatively unimportant compared to purging. Thus, when considering the consequences of inbreeding and purging on average fitness, predictions are reliable for *dN_e_* > 1, where *N_e_* is the drift effective population size (García-Dorado 2012). For panmictic populations of constant size, drift effective size is equal to inbreeding effective size (*N_e_* = 1/2Δ*F*, where Δ*F* is the per generation inbreeding rate), so that we can expect IP predictions to be reliable if, through the whole process, *d* > 2Δ*F*. This rate can be computed for consecutive time periods with length equal to the average generation interval. Thus, at each interval, Δ*F* = (*F*′−*F*)/(1−*F*), where *F* and *F*′ are the average inbreeding in the population at the beginning and the end of the interval.

### Data availability

The authors state that all data necessary for confirming the conclusions presented in the article are represented fully within the article. PURGd software and example data are available in https://www.ucm.es/genetica1/mecanismos.

## The Software

We present a new software package (PURGd, available from https://www.ucm.es/genetica1/mecanismos) that uses the IP model to jointly estimate the effective purging coefficient, *d*, and the inbreeding load in the base population, or its related parameter, *b*, defined in Equation 10, that better account for the fitness values of a set of pedigreed individuals. Additional details are given in the user’s guide included in the package.

The program computes standard coancestry and inbreeding (*f* and *F* values), as well as Ballou’s ancestral inbreeding coefficient (*F_a_*) for each individual. Furthermore, for each *d* value considered, it recurrently uses Equation 18, Equation 19 and Equation 22 to compute the corresponding purged inbreeding coefficients (*g*). Using these coefficients, the program obtains LS estimates for the *d* value, and for the remaining parameters in the model. As the predictive model may incorporate additional factors potentially affecting fitness, and since fitness is assumed to be a multiplicative trait, Equation 11 is generalized to include an arbitrary number of additional factors (say *x*, *z*…), giving the general model

$$E\left(W_{i}\right)=W_{\max}\left(1-\varepsilon_{i}\right)e^{\beta_{1}\;g_{i}+\beta_{2}\;x_{i}+\beta_{3}\;z_{i}...},$$ (23)

where *β*_1_ = −*δ* is the regression coefficient on purged inbreeding *g*, *g* is a function of *d*, and the remaining *β_j_* values measure the effect of the corresponding additional factors, which may include the maternal purged inbreeding coefficient.

This software numerically searches for the *d* value that minimizes the squared deviations from observed fitness to model predictions (*i.e.*, for the LS estimate). However, regarding the remaining parameters, the model can be fitted using two different approaches, as explained below. In the first approach (linear regression method, LR), for each *d* value considered, a LR model is fitted for log-transformed fitness. In the second approach (numerical nonlinear regression method, NNLR), the above model for untransformed fitness (Equation 23) is explored numerically, searching for the joint numerical LS estimates of *d* and of the nonlinear regression coefficients. Although the NNLR method is computationally more demanding, the program runs quickly, and has low RAM requirements under both approaches. Optionally, the initial average for fitness or log-fitness and/or for the regression coefficient on *g* can be introduced by the user, allowing incorporation of independent estimates of these parameters when available.

Additionally, the software will also give the results for the corresponding analysis conditional to *d* = 0, so that the user can observe the consequences of considering/ignoring purging in the analysis, and can check how the model improves under the estimate of *d*, compared to the assumption of no purging (*d* = 0).

### LR method

To perform LR analysis, the model represented by Equation 23 is linearized by taking logarithms. This leads to the linear predictive equation

$$\ln\left(W_{i}\right)=b_{0}+b_{1}\;g_{i}+b_{2}\;x_{i}+b_{2}\;z_{i}\ldots .,$$

where the different *b* values estimate the corresponding regression coefficients. Since logarithms are taken for individual fitness, instead of for average fitness, by analogy to Equation 7, the intercept *b*_0_ estimates *E*[ln(*W*_0_)], and *b*_1_ estimates [ln(1−2*d*)/2*d*]*δ* (Equation 10).

However, as it has been noted (García-Dorado 2012), the IP model is a conservative approach that tends to underrate the long-term fitness expected from inbreeding and purging. For this reason, when the estimate of the expected log-fitness for noninbred individuals (*b*_0_) is obtained jointly with *b*_1_ and with the purging coefficient (*d*), the method tends to overfit the model by estimating too low an initial fitness, and, simultaneously, too small values for the decline of log fitness with *F_i_* (*i.e.*, for −*b*_1_) and for the purging coefficient *d*. Thus, this procedure tends to give *b*_1_ and *d* estimates that will produce poor predictions when extrapolated to populations with different rates of inbreeding, or to periods of different length. On the contrary, when *E*[ln(*W*_0_)] is not simultaneously estimated, the estimates *b* and *d* have much smaller bias and good predictive properties.

Therefore, *b*_0_ is obtained by PURGd in a previous step as the average of log-fitness for noninbred individuals with noninbred ancestors (*F* = *F_a_* = 0), or is introduced by the user as a known value. Then, in a second step, the software searches for the *d* value that optimizes the fitting of the data to the linear regression equation

$$Y_{i}=b_{1}\;g_{i}+b_{2}\;x_{i}+b_{2}\;z_{i}\ldots .\;,$$

where the dependent variable is *Y_i_* = ln(*W_i_*) – *b*_0_, so that regression is forced through the origin.

Regression analysis is performed for all the possible *d* values in a grid corresponding to the interval 0 ≤ *d* ≤ 0.5 with step 0.01, which is the default accuracy. If higher accuracy is requested, PURGd first finds a preliminary estimate with precision 0.01 as before, and then uses the Golden Section Search (GSS) algorithm in an interval ± 0.01 around that estimate (Press *et al.* 1992).

Finally, the software returns the *d* estimate that minimizes the residual sum-of-squares in the corresponding LR analysis of individual log-fitness. For each analysis, the program also gives the corresponding results of the above LR, with statistic contrasts assuming normality and independence of residual errors, and with the adjusted determination coefficient and the corrected Akaike information criterion, computed taking into account how many parameters are being estimated in the whole process.

Table 1 reproduces the software’s output for the LR approach, where estimates have been averaged for the analysis of a set of 50 simulated lines. Each line is derived from a large panmictic population at the Mutation-Selection-Drift balance (*N* = 1000), and is maintained with size *N* = 10 during 50 generations. Completely recessive deleterious mutations with homozygous effect *s* = 0.3 occur at a rate of 0.1 new mutations per gamete and generation in unlinked sites. Since *h* = 0, this implies that the theoretical value for the purging coefficient is *d* = 0.15. The simulation details can be found in Bersabé *et al.* (2016). Output is presented for two different simulation sets; in the first, natural selection is operating during the maintenance of the lines, so that purging is expected to occur. In the second set, natural selection is relaxed, implying no purging. To achieve this, when simulating each offspring, all individuals had the same probability of being sampled as parents of the next generation, regardless of their fitness values. The software estimates a purging coefficient *d* = 0.102 ± 0.009 in the first case, and *d* = 0.003 ± 0.001 in the second (SE computed from 50 replicates). Therefore, the method has discriminated between situations with or without purging, although it has underestimated the actual purging coefficient. Furthermore, for lines undergoing purging, the data fit the IP model prediction computed using the corresponding estimate of *d* much better than when using the condition *d* = 0 that assumes no purging, as shown by the higher determination coefficient and the smaller residual sum of squares and Akaike criterion.

**Table 1:** Averaged results obtained using the linear regression method (LR) for the set of 50 simulated lines described in the main text that were maintained with size *N* = 10 during 50 generations, where the true values for the inbreeding load and the purging coefficients in the base population are δ = 4.217 and *d* = 0.15, respectively

Pedigree File	Analysis	*d* Coefficient	RSS	*P*-Value (*F*)	*aR2*	AICc	ln*W*_0_	SD(ln*W*_0_)	*b*(*g*)	SD[*b*(*g*)]	*P*-Value(*t*)
Purged_lines	IP model	0.102	147.291	<1.0e−16	0.758	804.642	−0.124	0.206	−3.298	0.081	<1.0e−16
	No-purging model	0	253.130	<1.0e−16	0.586	1069.500	−0.124	0.206	−1.222	0.041	<1.0e−16
Relaxed_lines	IP model	0.003	188.396	<1.0e−16	0.966	921.812	−0.122	0.201	−5.177	0.040	<1.0e−16
	No-purging model	0	195.72	<1.0e−16	0.964	944.204	−0.122	0.201	−4.965	0.039	<1.0e−16

These results are shown in the same format as in the PURGd output. Pedigree File, name of the data file; Analysis, the model used in the analysis; *d* coefficient, the purging coefficient estimated in the IP analysis or assumed by the No-purging model; RSS, residual sum of squares; *P*-value(*F*), the *P*-value in the *F*-test for the regression analysis; *aR2*, adjusted determination coefficient; AICc, the corrected Akaike Information Criterion; ln*W*_0_, the estimate of the expected log-fitness in the base noninbred population; SD(ln*W*_0_), SD of ln*W*_0_; *b*(*g*), linear regression coefficient on *g* (it is denoted *b*_1_ in the predictive equation and estimates [ln(1−2d)/2d]*δ*, as defined in Equation 10; its expected value in this case is −5.014, very close to the IP estimate obtained for the relaxed lines); SD[*b*(*g*)], SD of *b*(*g*); *P*-value(*t*), *P*-value for the *t*-test on the significance of this linear regression coefficient.

The analysis of additional simulated lines maintained with size *N* = 50 (not shown) produced similar results, again discriminating between purged and relaxed lines and providing better fitting for purged lines when using the corresponding estimates of *d*. For purged lines, the estimate for the regression coefficient of fitness on purged inbreeding was *b*(*g*) = −3.590 ± 0.276 which, solving Equation 10, gives an estimate *δ* = 3.019 for the inbreeding load, close to the value obtained for *N* = 10 (*δ* = 2.774), but the estimate for the purging coefficient was larger (*d* = 0.218 ± 0.029).

### NNLR method

The previous logarithmic transformation cannot be applied to fitness traits presenting null values, as in the case binary of 0/1 variables for dead/alive records. In such cases, inbreeding depression has been analyzed previously using a logit transformation of fitness in order to perform multiple logistic regression (Ballou 1997; Boakes and Wang 2005). However, that statistical approach assumes a model of the kind ln[*W_i_*/(1−*W_i_*)] = β_0_ − β_1_ *g_i_*, while our genetic model has the form ln(*W_i_*) = β_0_ − β_1_ *g_i_*. Therefore, PURGd gives the user the option of obtaining LS estimates for the parameters in the genetic model given by Equation 23 by numerically optimizing the fitting of the untransformed fitness data to the predictions of the nonlinear regression equation given by

$$W_{i}=W_{0\;}e^{b_{1}g_{i}+b_{2}\;x_{i}+b_{3}\;z_{i}\ldots },$$

where the different *b* values are the estimates of the corresponding β parameter in Equation 23, so that *b*_1_ estimates −*δ*, and *W*_0_ is the estimate of the expected fitness value for the noninbred base population. For the same reasons as in the LR method, *W*_0_ is obtained in a previous step as the average *W* for the set of individuals with *F* = *F_a_* = 0, or is introduced by the user.

After estimating *W*_0_, the Numerical Least Square option of PURGd uses the Artificial Bee Colony (ABC) algorithm (Karaboga and Basturk 2007) to search simultaneously for the LS estimate of the purging coefficient *d* (where each *d* value considered determines a set of *g_i_* values), and for the set of *b* coefficients that produces the lowest residual sum of squares (RSS), calculated as:

$$\mathrm{RSS=}\sum_{i}{\left(W_{i}-W_{0}\;e^{b_{1}\;g_{i}+b_{2}\;x_{i}+b_{3}\;z_{i}\ldots }\right)}^{2}$$

This algorithm has been used successfully for estimating parameters in nonlinear systems in different kinds of disciplines, such as image processing, engineering, and neural networks, among others (Karaboga *et al.* 2014), using ∼500 generations and 250 bees in the colony. Although we have always found consistent solutions, it is recommended to repeat the analysis several times to check the stability of the method, and to change running parameters and range values, looking for a consistent solution.

Therefore, the output gives a LS estimate for *d*, and for the remaining $\beta_{j}$ parameters in the model (Equation 23). An important advantage of this approach is that, besides allowing 0 fitness values to be dealt with, −*b*_1_ directly estimates the inbreeding load *δ*, instead of estimating −[ln(1−2*d*)/2*d*]*δ*. Furthermore, although LS estimates for nonlinear regression are not expected to be unbiased, preliminary unpublished simulated results suggest that this method usually gives estimates of the purging coefficient and of the inbreeding load that produce predictions at least as accurate as those obtained using estimates computed from linear regression on log-fitness data, although it is computationally more demanding. Although this approach does not allow standard *F*-tests for statistical significance to be performed, the RSS and the corrected Akaike information criterion values (the latter again relying on the assumption of normality and independence for residual errors) are reported in the output as a measure of the fitting quality.

Table 2 reproduces the software’s output for this NNLR approach, where estimates have been averaged for analysis of the same sets of simulated lines analyzed in Table 1. In this case, the estimates of the purging coefficient for lines maintained with natural selection is *d* = 0.092 ± 0.007, and that obtained for lines maintained under relaxed selection is *d* = 0.007 ± 0.001, again discriminating between purging and no purging cases, but underestimating the purging coefficient (SE again empirically estimated from the 50 replicated lines). As in the LR method, the data for simulated lines undergoing purging fit the IP model much better than the *d* = 0 no-purging model.

**Table 2:** Averaged results obtained using the numerical nonlinear regression method (NNLR) for the set of 50 simulated lines described in the main text that were maintained with size *N* = 10 during 50 generations, where the true values for the inbreeding load and the purging coefficients in the base population are δ = 4.217 and *d* = 0.15, respectively

Pedigree File	Analysis	*d* Coefficient	RSS	AICc	*W*_0_	SD(*W*_0_)	*b*(*g*)
Purged_lines	IP model	0.092	16.996	−326.399	0.902	0.152	−2.898
	No- purging model	0	28.387	−71.356	0.902	0.152	−1.202
Relaxed_lines	IP model	0.007	4.072	−1037.943	0.903	0.154	−4.533
	No-purging model	0	4.145	−1033.899	0.903	0.154	−4.443

These results are shown in the same format as in the PURGd output. Pedigree File, name of the data file; Analysis, the model used in the analysis; *d* coefficient, the purging coefficient estimated in the IP analysis or assumed by the No-purging model; RSS, residual sum of squares; AICc, the corrected Akaike Information Criterion; *W*_0_, the estimate of the expected fitness in the base noninbred population; SD(*W*_0_), SD of *W*_0_; *b*(*g*), nonlinear regression coefficient on *g* that estimates the inbreeding load (*b*(*g*), denoted *b*_1_ in the predictive equation, estimates −*δ*).

For simulated lines maintained with size *N* = 50 (not shown), NNLR analysis of the data discriminated between purged and relaxed lines, and provided better fitting for purged lines when using the corresponding estimates of *d*, as in the case of the LR analysis. Again, the estimate for the inbreeding load for purged lines (*δ* = −*b*(*g*) = 2.756 ± 0.241), was very close to that estimated for *N* = 10, but the estimate for the purging coefficient was larger (*d* = 0.190 ± 0.005).

### Predictive value of the estimates

**Figure 2:**
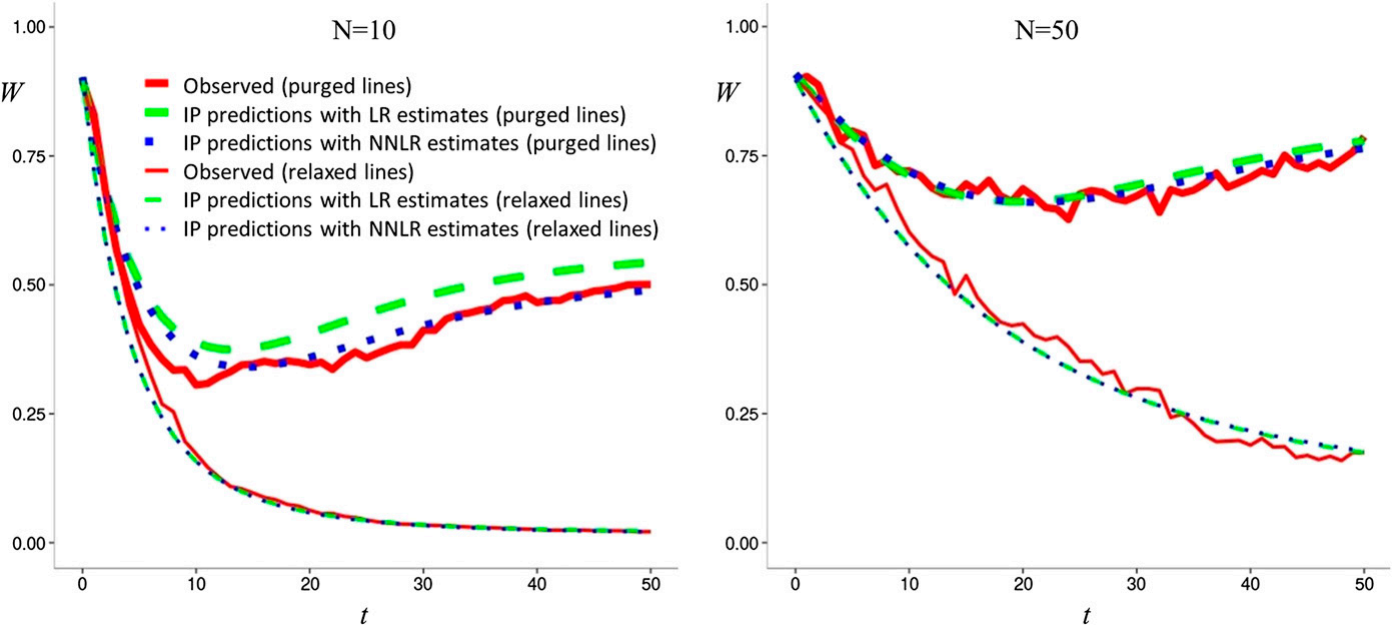
Evolution of mean fitness through generations for simulated lines maintained with size *N* = 10 (analysis given in Table 1 and Table 2) or *N* = 50 during 50 generations (red solid lines), together with IP predictions computed using the estimates obtained by PURGd from the linear regression method (LR, green dashed lines), or the numerical nonlinear regression method (NNLR, blue dotted lines). Results are given both for lines that have undergone purging (thick lines), and for lines for which natural selection was relaxed while they were maintained with reduced size (thin lines, which largely overlap with each other).

Figure 2 gives the evolution of fitness against generation number and the corresponding IP predictions, computed for each set of lines using, in Equation 12, the corresponding estimates of *δ* and *d* obtained by the software. Good fitting is observed for *N* = 10 and for *N* = 50 regardless of whether LR or NNLR are used, both for the relaxed lines and for those maintained under purging.

## Discussion

In the present work, we derive a theoretical approach to analyze the fitness data for pedigreed individuals in order to estimate the inbreeding load, *δ*, and the purging coefficient, *d*, necessary to predict the joint consequences of inbreeding and purging. Furthermore, we present PURGd, a free software implementing this theoretical approach, and illustrate its performance, analyzing some results obtained by the software for simulated data.

In the first place, since the inbreeding depression rate is usually estimated from log-fitness data, we derive the expected regression slope of individual log-fitness on individual inbreeding in the absence of selection, which amounts to *b* = [ln(1−2*d*)/2*d*]*δ*. Therefore, using –*b* as an estimate of the inbreeding load, *δ*, implies upwardly biased estimation. This first result is interesting because increased effort in field studies related to conservation of endangered species, together with molecular techniques, allow us to record and/or reconstruct pedigrees in wild populations, and offers an interesting opportunity to study inbreeding depression in the wild (Keller and Waller 2002), but can induce upwardly biased estimates due to the use of log-transformed individual fitness. The bias is expected to be small if *d* values are low, but the large inbreeding depression rates estimated in wild populations are likely to be associated with relatively large *d* values and, therefore, to substantial bias (Kruuk *et al.* 2002; Liberg *et al.* 2005; O’Grady *et al.* 2006; Walling *et al.* 2011; Kennedy *et al.* 2014; Hedrick *et al.* 2016). This phenomenon can contribute to enhancing the perceived difference between the inbreeding load expressed in wild populations compared to estimates based on the assay of mean fitness for groups of individuals with different average inbreeding, as is often the case in experimental conditions. In order to avoid this bias, an alternative estimation approach is suggested, based on the numerical LS analysis of the original predictive IP model for untransformed fitness. This approach is implemented in the PURGd software, and is used to analyze some simulated data.

In the second place, in order to estimate the purging coefficients (*d*) from individual fitness data, we present general expressions to compute purged inbreeding (*g*) from pedigrees with overlapping generations. Although these expressions involve some approximations, we have found that they produce reliable values for individual *g*.

Other methods for detecting purging from fitness measured in pedigreed individuals have been devised previously, based on the idea that the ancestral purging *F_a_* of an individual is in some way related to the opportunities of purging upon its genome in previous generations. Using *F* and *F_a_*, different linear models have been proposed that have, on some occasions, detected small levels of purging in simulated and real pedigrees of captive breeding populations (Ballou 1997; Lacy and Ballou 1998; Boakes and Wang 2005; Swindell and Bouzat 2006; Boakes *et al.* 2007; Ceballos and Álvarez 2013). However, these methods were based on the analysis of statistical models that are not supported by a predictive genetic model. In addition, a logit transformation was applied to fitness, just on statistical grounds. Therefore, these models fit fitness data only poorly. More importantly, they do not allow estimation of a purging parameter that can be used for predictive purposes. On the contrary, our method is based on the predictive IP model that was derived on the basis of the genetic mechanisms of inbreeding depression and purging, so that it is expected to fit the data better, and to allow the estimation of a parameter that can be used for predictive purposes: the effective purging coefficient *d*. However, the model involves some approximations and usually produces conservative predictions underrating the consequences of purging. Therefore, statistical methods based on this IP model can overfit the model by inducing some bias in the estimates.

For illustrative purposes, we have presented here the analysis of a set of simulated data for a simple situation where inbreeding and purging occur due to a reduction in population size (Table 1 and Table 2). For *N* = 10, the inbreeding load computed using Equation 1 in the base simulated population was *δ* = 4.217. The LR method estimates *d* = 0.102 ± 0.009 and *b* = −3.298 ± 0.096 (SE computed from the 50 replicates analyzed), which using the true simulated value for *d* (0.15) into Equation 6 gives an estimate of the inbreeding load of *δ* = 2.774. Thus, both the inbreeding load and the purging coefficient are underestimated when they are estimated jointly. The *δ* and *d* estimates obtained using the numerical method are very similar (2.898 ± 0.115 and 0.092 ± 0.007, respectively). Under both methods, the data fit the IP model much better than the no-purging (*d* = 0) model. In parallel, we present the analysis for a similar set of simulated lines where selection, and, therefore, purging, had been relaxed during the inbreeding period. It is worth noting that the estimates of the purging coefficient *d* given by PURGd for these relaxed lines are virtually zero, showing that the method detects whether purging is occurring or not. Furthermore, when natural selection is relaxed during the maintenance of the reduced size lines, the LR approach gives *b* = −5.177 ± 0.165, so that the estimate of *δ* is 4.354, and the *δ* estimate obtained using the numerical approach is very similar (4.533). Thus, the underestimation of *δ* observed when purging is operating in the lines, can be ascribed to regression overfitting the data through the underestimation of both *δ* and *d*, due to the approximate nature of the IP model. It should be noted that some underestimation of *d* could also occur because, for *Nd* on the order of 1 or smaller, purging efficiency may be somewhat reduced due to genetic drift (García-Dorado 2012). On the contrary, *d* estimates obtained for simulated purged lines maintained with *N* = 50 are larger than the actual *d* value, while *δ* is simultaneously underestimated. In all cases, using the *δ* and *d* estimates obtained jointly in the same analysis gives appropriate predictions for the evolution of mean fitness (Figure 2).

The software also allows additional factors, both in the linear and the nonlinear models, to be included. However, the addition of factors with a strong association with *g*, as maternal inbreeding or year of birth, often causes a slight overfitting, again due to the approximate nature of the program. The overfitted model gives spurious significant effects for such factors as well as some distortion in the estimates of *b*(*g*) and *d* (results not shown) due to confounded effects. Therefore, results obtained by incorporating additional factors should better be used when those factors are uncorrelated to *g*, so that including them just reduces sampling error. Additional factors should also be tentatively included when there is external evidence that they have a highly relevant effect, so that including them cause an important improvement of the fitting statistics. However, when these additional factors are correlated to *g*, these results should be interpreted with caution, and those obtained including no additional factors should also be considered.

It is interesting to note that using, in Equation 12, the estimates of *δ* and *d* obtained by the software, produces predictions that adequately fit the evolution of mean fitness through generations in the simulated lines, both in the absence and in the presence of purging (Figure 2).

Summarizing, we present a version of the IP model that analyzes individual fitness data for pedigreed individuals, is able to detect purging, and estimates genetic parameters that are useful in predicting the joint consequences of inbreeding and purging. However, it is necessary to explore the properties of this approach more extensively through the analysis of simulated data with different rates of inbreeding, and with different distributions of the *h* and *s* values of deleterious mutations. Furthermore, it would be useful to compare its performance with that of previous methods based on ancestral inbreeding, and to characterize the possible biases of our method regarding the estimates of *d* and *δ* caused by the approximate nature of our IP model, as well as their predictive implications. This exploration needs to analyze a wide range of simulated situations, including different population sizes, generation numbers, and distributions of the deleterious effects, and will be addressed in a different paper.
